# Glucose absorption and gastric emptying in critical illness

**DOI:** 10.1186/cc8021

**Published:** 2009-08-27

**Authors:** Marianne J Chapman, Robert JL Fraser, Geoffrey Matthews, Antonietta Russo, Max Bellon, Laura K Besanko, Karen L Jones, Ross Butler, Barry Chatterton, Michael Horowitz

**Affiliations:** 1 Department of Anaesthesia and Intensive Care, Royal Adelaide Hospital, North Terrace, Adelaide, SA 5000, Australia; 2 School of Medicine, University of Adelaide, Adelaide, SA 5000, Australia; 3 Investigation & Procedures Unit, Repatriation General Hospital, Daws Road, Daw Park, SA 5041, Australia; 4 Centre for Paediatric and Adolescent Gastroenterology, Women's and Children's Hospital; 72 King William Road, Adelaide, SA 5006, Australia; 5 Department of Nuclear Medicine, Royal Adelaide Hospital, North Terrace, Adelaide, SA 5000, Australia

## Abstract

**Introduction:**

Delayed gastric emptying occurs frequently in critically ill patients and has the potential to adversely affect both the rate, and extent, of nutrient absorption. However, there is limited information about nutrient absorption in the critically ill, and the relationship between gastric emptying (GE) and absorption has hitherto not been evaluated. The aim of this study was to quantify glucose absorption and the relationships between GE, glucose absorption and glycaemia in critically ill patients.

**Methods:**

Studies were performed in nineteen mechanically-ventilated critically ill patients and compared to nineteen healthy subjects. Following 4 hours fasting, 100 ml of Ensure, 2 g 3-O-methyl glucose (3-OMG) and ^99m^Tc sulphur colloid were infused into the stomach over 5 minutes. Glucose absorption (plasma 3-OMG), blood glucose levels and GE (scintigraphy) were measured over four hours. Data are mean ± SEM. A *P*-value < 0.05 was considered significant.

**Results:**

Absorption of 3-OMG was markedly reduced in patients (AUC_240_: 26.2 ± 18.4 vs. 66.6 ± 16.8; *P *< 0.001; peak: 0.17 ± 0.12 vs. 0.37 ± 0.098 mMol/l; *P *< 0.001; time to peak; 151 ± 84 vs. 89 ± 33 minutes; *P *= 0.007); and both the baseline (8.0 ± 2.1 vs. 5.6 ± 0.23 mMol/l; *P *< 0.001) and peak (10.0 ± 2.2 vs. 7.7 ± 0.2 mMol/l; *P *< 0.001) blood glucose levels were higher in patients; compared to healthy subjects. In patients; 3-OMG absorption was directly related to GE (AUC_240_; r = -0.77 to -0.87; *P *< 0.001; peak concentrations; r = -0.75 to -0.81; *P *= 0.001; time to peak; r = 0.89-0.94; *P *< 0.001); but when GE was normal (percent retention_240 _< 10%; n = 9) absorption was still impaired. GE was inversely related to baseline blood glucose, such that elevated levels were associated with slower GE (ret 60, 180 and 240 minutes: r > 0.51; *P *< 0.05).

**Conclusions:**

In critically ill patients; (i) the rate and extent of glucose absorption are markedly reduced; (ii) GE is a major determinant of the rate of absorption, but does not fully account for the extent of impaired absorption; (iii) blood glucose concentration could be one of a number of factors affecting GE.

## Introduction

Delayed gastric emptying (GE) occurs frequently in critically ill patients [1] and is associated with impaired tolerance to naso-gastric feeding [2]. By slowing the transfer of food from the stomach into the small intestine and, thereby, reducing or delaying exposure of nutrient to small bowel mucosa, gastric stasis has the potential to adversely affect both the rate and extent of nutrient absorption [3]. Absorption may also be compromised by factors other than GE, including the rate of small intestinal transit, mucosal villous atrophy or oedema and reduced splanchnic perfusion. There is limited information about nutrient absorption in critically ill patients, and the relation between GE and absorption has hitherto not been evaluated.

Postprandial blood glucose concentrations are affected by many factors, including GE and small intestinal glucose absorption [3,4]. In health, the relation between GE and glycaemia is complex. Acute hyperglycaemia, including elevations in blood glucose that are within the normal postprandial range, has been shown to slow GE when compared with euglycaemia [5]. However, a reduced rate of GE will also slow the rate of carbohydrate absorption [6] and, thereby, attenuate the rise in blood glucose following a carbohydrate meal [3,7]. Thus, in health and in type 2 diabetes, the rate of GE is both a determinant of, as well as being determined by, blood glucose concentrations [4]. The relation between glycaemia and GE in critically ill patients has hitherto not been evaluated. Hyperglycaemia is usually attributed to insulin resistance and elevated glucagon concentrations, which frequently occur even when there is no history of diabetes [8]. This could contribute to the delayed GE observed in many critically ill patients. Conversely, delayed GE may potentially attenuate hyperglycaemia in patients fed by the naso-gastric route. There is evidence that maintenance of blood glucose concentrations in the euglycaemic range improves outcomes in critically ill patients [9]. Hence, an improved understanding of the factors influencing glycaemia is important.

The aims of this study were to quantify glucose absorption and assess the relations between absorption and glycaemia with GE in critically ill patients.

## Materials and methods

### Subjects

Nineteen mechanically ventilated critically ill patients, who were receiving or eligible to receive naso-gastric nutrition, were recruited from a mixed medical/surgical intensive care unit (ICU). The study was approved by the Research Ethics Committee of the Royal Adelaide Hospital and performed in accordance with NH&MRC guidelines for research involving critically ill humans. In all cases, critically ill patients were unable to provide their own consent and written informed consent was obtained from their next of kin. Exclusion criteria were (i) pre-existing diabetes mellitus, (ii) contraindication to placement of a naso-gastric tube, (iii) oesophageal, gastric or duodenal surgery within the previous three months, and (iv) pregnancy/lactation. Three patients were receiving short-acting insulin during the study period for control of hyperglycaemia and were excluded from the evaluation of blood glucose concentrations (leaving 16 subjects for blood glucose data analysis). Prokinetic drugs were withheld during the study period. The patients remained on the sedative regimen that they were receiving as part of their ICU care. In the majority of cases, this was a combination of morphine and midazolam given as a continuous infusion.

The patient data were compared with 19 healthy volunteers. Healthy subjects provided written, informed consent prior to participating in the study.

### Protocol

#### Healthy subjects

Healthy volunteers were studied in the morning, after an overnight fast. A naso-gastric tube was inserted for the purpose of the study and its correct positioning was verified by measuring pH aspirates and auscultation of air infusion.

#### Critically ill patients

Critically ill patients were studied in the morning, after a fast of at least four hours. In all cases, a naso-gastric tube was *in situ *prior to the study. Correct tube positioning was confirmed radiologically and by measurement of pH aspirates prior to commencing the study.

Following aspiration of the naso-gastric tube, 100 ml of Ensure (Abbott laboratories BV, Zwolle, Holland - standard liquid feed - 1 kcal/ml) combined with 2 g of 3-O-methyl glucose (3-OMG) (Sigma-Aldrich Pty. Ltd. Castle Hill, NSW, Australia) and labelled with ^99m^Tc sulphur colloid (Royal Adelaide Hospital radiopharmacy, Adelaide, South Australia), was infused into the stomach over five minutes. Following test meal delivery (Time = 0), scintigraphic measurements of GE (see below) were performed over four hours. Blood samples were obtained at timed intervals during the study for the measurement of blood glucose and plasma 3-OMG concentrations (see below).

#### Glucose absorption

Glucose absorption was measured using 3-OMG, a previously validated technique [10]. Plasma 3-OMG concentration was quantified on arterial (critically ill patients) or venous (healthy subjects) blood samples at baseline and at 5, 15, 30, 45, 60, 90, 120, 150, 180, 210 and 240 minutes and analysed by high performance exchange chromatography [11]. Data were assessed for peak and time to peak 3-OMG concentration and areas under the curve at 240 minutes (AUC_240_).

#### Blood glucose concentrations

Blood glucose concentrations were measured using a bedside glucometer (MediSense Precision, Abbott Laboratories, MediSense Products, Bedford, MA, USA), using arterial (critically ill patients) or venous (healthy subjects) samples at baseline and at 5, 15, 30, 45, 60, 90, 120, 150, 180, 210 and 240 minutes. Glucose data was assessed for baseline level, peak, time to peak and change in concentration from baseline.

#### Gastric emptying

GE was measured using scintigraphy. In critically ill patients, this was performed in the ICU using a mobile gamma camera (GE Starcam 300 AM General Electric (Milwaukee, Wisconsin, USA) - with three-minute dynamic frame acquisition). Healthy subjects were studied in the Department of Nuclear Medicine, PET & Bone Densitometry, Royal Adelaide Hospital, using a single-headed, stationary, gamma camera (GE millennium MPR Cardiff, UK) with data acquisition in three-minute frames. Reframed data were corrected for subject movement and radionuclide decay and scatter. All subjects were studied for four hours supine, in the 20° left anterior oblique position [12]. A gastric region-of-interest was identified and used to derive GE curves (expressed as percent of the maximum content of the total stomach). The intragastric content at 60, 120, 180 and 240 minutes was determined [13].

### Statistical analysis

Data are shown as mean values ± standard deviation, or median and range, as appropriate. Statistical analysis was performed using SPSS version 14.0 (SPSS Inc, Chicago, Illinois, USA) or Minitab 13 for windows (Minitab Inc, State College, PA, USA). The distribution of data was determined using D'Agostino Pearson omnibus test. Differences between normally distributed data were analysed using Student's t test. Data not normally distributed were analysed using the Mann-Whitney U test. In studies where a number of measures were performed over time, a repeated analysis of variance was used to analyse the data. Correlations were performed using Pearson correlation coefficients. Normal ranges were defined as the range of values in the healthy cohort. For the analysis of the relation between glucose absorption and GE the natural log of the area under curve (AUC) values was used because of substantial heterogeneity in the data. This was examined using analysis of covariance and deviation from a regression line. Relations between glucose absorption (baseline level, peak concentration, time to peak and area under the 3-OMG concentration curve) and blood glucose with GE were examined. A *P *value of ≤ 0.05 was considered significant in all analyses.

## Results

The study was tolerated well by all subjects and there were no adverse events. Demographic information about critically ill patients and healthy subjects are summarised in Table 1. In two healthy subjects, blood sampling was not possible for the full four hours (60 minutes in one and 150 minutes in the other). Scintigraphic data were not available in one patient due to technical difficulties. In patients, the median gastric residual volume immediately prior to the study was 5 ml (range 0 to 120).

**Table 1:** Demographics of study participants

	ICU patients (n = 19)	Healthy subjects (n = 19)
**Age (years) (median range)**	63 (28 to 79)*	24 (21 to 51)
**Gender (M:F)**	14:5	7:12
		
**BMI (kg/m^2^)**	26 (20 to 38)	24.4 (20 to 34.5)
		
**APACHE II score (study day)**	16 (12 to 26)	N/A
		
**Diagnostic groups (n)**	Trauma (5) ICH (3) Sepsis (4) Respiratory failure (3) Vascular (2) Post-op ENT (1) Burns (1)	N/A
		
**Hospital survival (%)**	80	N/A
		
**Continuous renal replacement therapy (n)**	3	N/A
		
**Insulin therapy (n)**	3	N/A
		
**Baseline blood glucose level** **(mMol/L). Mean (SD)**	8.0 ± 2.1 *	5.6 ± 0.23
**Prokinetics prior to study (n)**	9	N/A
		
**Catecholamines (n)**	2	N/A
		
**Propofol (n)**	3	N/A

* *P *< 0.001 when patients compared with healthy subjects.

APACHE = acute physiology and chronic health evaluation; BMI = body mass index; ENT = ear nose and throat; F = female; ICH = intracranial haemorrhage; ICU = intensive care unit; M = male; Post-op = postoperative; SD = standard deviation.

### 3-OMG absorption

**Figure 1:**
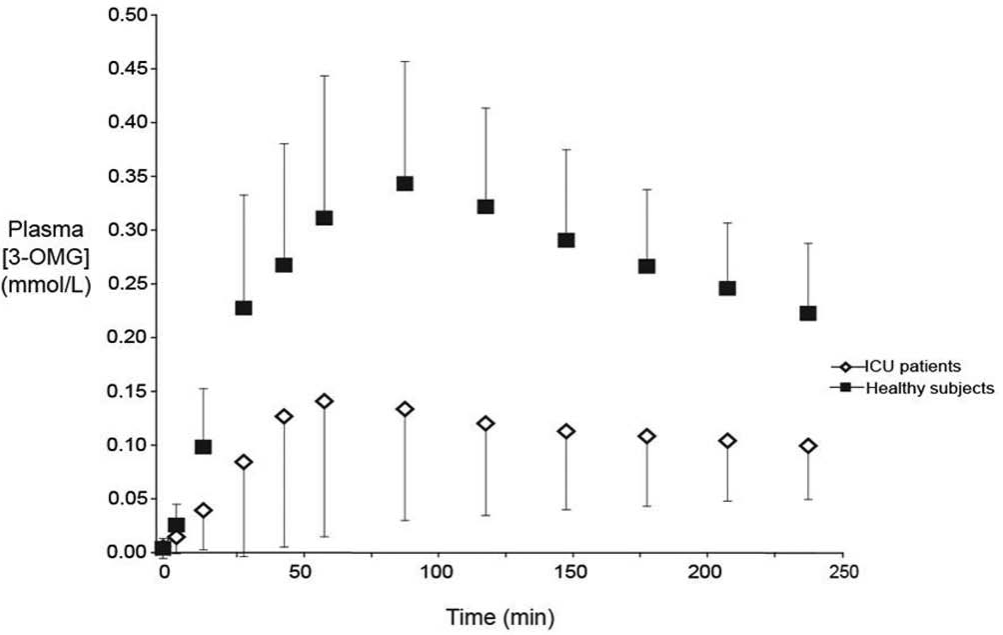
Plasma 3-OMG concentrations in ICU patients (n = 19) and healthy controls (n = 19). Area under the concentration curve at 240 minutes (AUC_240_): *P *< 0.001; Peak [3-OMG]: *P *< 0.001; Time to peak: *P *= 0.007. ICU = intensive care unit.

There was a significant increase in plasma 3-OMG in both groups (*P *< 0.001 for both) following the nutrient bolus. The 3-OMG AUC (AUC_240_: 26.2 ± 18.4 vs. 66.6 ± 16.8; *P *< 0.001), as well as the peak 3-OMG concentration AUC (0.17 ± 0.12 vs. 0.37 ± 0.098 mMol/l; *P *< 0.001) were markedly less in critically ill patients than healthy subjects (Figure 1). The time to peak was also longer in critically ill patients (151 ± 84 vs. 89 ± 33 minutes; *P *= 0.007), showing maximum 3-OMG concentration at 240 minutes for six patients (i.e. the end of the sampling period). Plasma 3-OMG had not returned to baseline at four hours in any subject.

### Blood glucose concentrations

**Figure 2:**
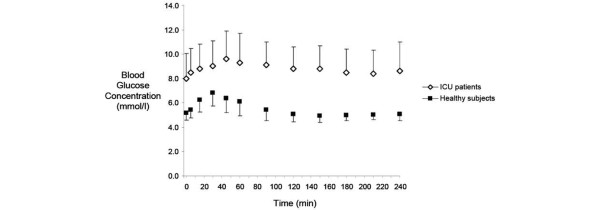
Blood glucose concentrations over time in ICU patients not receiving insulin (n = 16) and healthy subjects (n = 19). Peak blood glucose level was higher in the ICU patients (*P *< 0.001) with a delayed peak (*P *< 0.001). ICU = intensive care unit.

The baseline blood glucose level (8.0 ± 2.1 vs. 5.6 ± 0.23 mMol/l; *P *< 0.001) and peak concentration following nutrient administration (10.0 ± 2.2 vs. 7.7 ± 0.2 mMol/l; *P *< 0.001; Figure 2) were higher in critically ill patients compared with healthy subjects. The time to peak blood glucose was also longer in the critically ill patients (116 ± 90 vs. 39 ± 17 minutes; *P *< 0.001). There was no difference in the increment in blood glucose concentration following this dose of nutrient between the two groups.

### Gastric emptying

**Figure 3:**
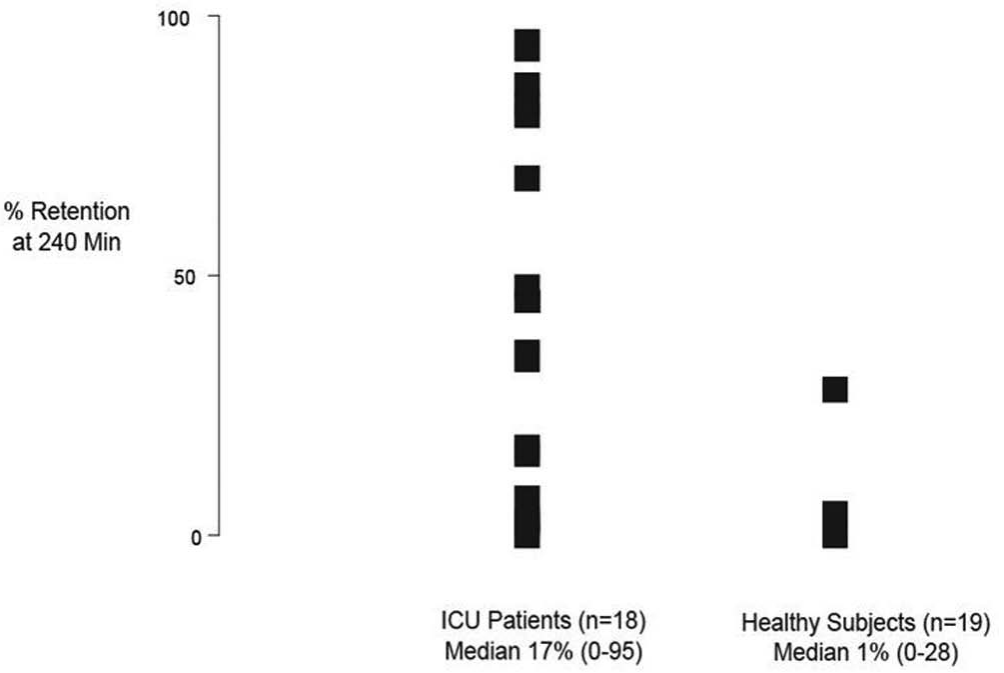
Gastric emptying (percent retention at 240 minutes) in ICU patients (n = 18) and healthy controls (n = 19). *P *< 0.05. ICU = intensive care unit.

GE data are shown in Figure 3. GE was slower in the critically ill patients compared with the healthy subjects (*P *= 0.024).

### Relations between 3-OMG absorption, blood glucose concentrations and gastric emptying

**Figure 4:**
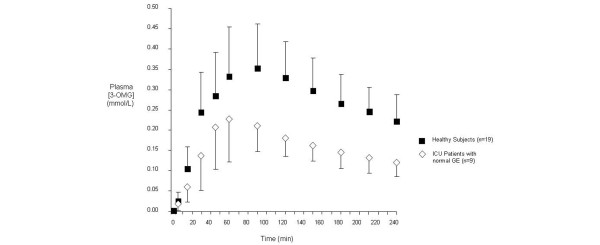
Plasma 3-OMG concentrations in ICU patients with normal GE (percent retention at 240 minutes <10%; n = 9) and healthy controls (n = 19). Area under the concentration curve at 240 minutes (AUC_240_): *P *< 0.001; Peak [3-OMG]: *P *= 0.006; Time to peak: *P *> 0.05. ICU = intensive care unit.

In critically ill patients, there was a close relation between all parameters of 3-OMG absorption (AUC_240_, peak concentration, time to peak) with GE (intra-gastric meal retention at all time-points). There was an inverse relation between plasma 3-OMG (AUC_240_; r = -0.77 to -0.87; *P *< 0.001; peak concentrations; r = -0.75 - -0.81; *P *= 0.001) and a positive relation between the time to peak 3-OMG concentration (r = 0.89-0.94; *P *< 0.001) with GE. In the healthy subjects, there was a significant relation between time to peak 3-OMG concentration and GE (retention at 60 minutes r = 0.64; *P *= 0.004; retention at 120 minutes r = 0.75; *P *< 0.001). In the subset of patients with normal gastric emptying (<10% retention at 240 minutes; n = 9), 3-OMG absorption was still less than in the healthy subjects (AUC_240_: 38.9 ± 11.4 vs. 66.6 ± 16.8; *P *< 0.001; Figure 4). In this subgroup, maximum 3-OMG concentration was also less than in healthy subjects (0.25 ± 0.09 vs. 0.37 ± 0.098 mmol/l; *P *= 0.006); but there was no difference in the time to maximum concentration (80 ± 36 vs. 89 ± 34 minutes; *P *> 0.05).

GE was inversely related to the baseline blood glucose level in the 16 critically ill patients who were not receiving insulin (retention at 60, 180 and 240 minutes - %; r = 0.51 to 0.54; *P *< 0.05). There was no significant relations between peak, time to peak or increment in blood glucose concentrations with GE. In the healthy subjects, there was no significant relation between GE and blood glucose at baseline. However, there was a weak relation between the change in blood glucose with GE, such that the increment in blood glucose was less when GE was slower (e.g. blood glucose increment vs. percent retention at 60 minutes, r = -0.45; *P *= 0.04).

There was no significant relation between 3-OMG absorption and baseline blood glucose in either the healthy subjects or critically ill patients. However, in the critically ill patients there was a relation between the increment in blood glucose and 3-OMG (AUC _240 _r = 0.70, *P *= 0.004; peak 3-OMG r = 0.73, *P *= 0.002; time to peak 3-OMG r = -0.62; *P *= 0.01). In the healthy subjects, there was a relation between time to peak blood glucose and time to peak 3-OMG concentration and (r = 0.52; *P *= 0.001).

## Discussion

This study suggests that both the rate and extent of glucose absorption are markedly reduced in critically ill patients [14-16], and demonstrates that there is a close relation between glucose absorption and GE in these patients, such that slow GE is associated with a reduced rate of absorption. An important new finding is that, even when GE is normal, glucose absorption is impaired. This indicates that there are additional causes to account for impaired absorption, other than delayed GE. A relation was also demonstrated between the increment in plasma glucose after the nutrient bolus and glucose absorption.

Two authors have previously reported reduced sugar absorption in critically ill patients. Singh and colleagues [16] found that plasma xylose concentrations were markedly reduced one hour after administration in patients with severe sepsis and trauma [16]. Similarly, measuring a single plasma level at 120 minutes, Chiolero and colleagues [14] demonstrated reduced, or delayed, absorption of xylose in a mixed group of ICU patients [14]. These studies did not attempt to differentiate between rate and total absorption. The rate of absorption is indicated by the time taken to reach maximum concentration in the blood [17], the maximum concentration achieved after a dose of substrate reflects both of these factors. The total absorption is indicated by the AUC, which reflects the extent of substrate absorbed over that time period [17].

Our study confirms that the rate of glucose absorption is reduced in critical illness. It also suggests that total absorption is reduced. This result needs further confirmation as 3-OMG concentrations had not returned to baseline at the end of the four-hour period. So it is possible that had the blood sampling continued, complete absorption may have eventually occurred; however, this appears unlikely. Hadfield and colleagues [15] assessed total 3-OMG absorption in a critically ill cohort by measuring urinary concentrations and found it to be reduced to approximately 20% of normal [15]. Accordingly, although we could only calculate AUC 0 to 240 minutes, it is likely that both rate and total glucose absorption are affected. However, when the rate of GE is normal the rate of glucose absorption also appears to be normal, even though total absorption may be reduced.

Glucose absorption across enterocytes takes place predominantly in the proximal small intestine, via the sodium-glucose cotransporter (SGLT 1) at the luminal membrane and the GLUT2 at the basolateral membrane [18]. Increased blood glucose concentrations are associated with increased glucose absorption [19]. In the rat, hyperglycaemia increases glucose uptake by increasing the activity of intestinal disaccharidases [20] and the number or activity of carriers at the basolateral membrane [21]. In the current study no relation was observed between baseline blood glucose concentrations and glucose absorption.

The rate and/or extent of glucose absorption is dependent on a number of factors that include GE, the presence of pancreatic enzymes, contact time with the small intestinal mucosa (transit), contact surface area (length of intestine, surface villi, enzyme content of brush border, and function of carrier molecules) and the depth of the diffusion barrier of the absorptive epithelium (unstirred layer) [22]. The underlying causes of the probable reductions in total glucose absorption in critical illness are unclear. Although in this study there was a relation between the rate of glucose absorption and GE, this did not account for the reduction in total absorption. Small intestinal mucosal abnormalities are known to occur in critically ill patients and are likely to be an important cause of malabsorption. Villous height and crypt depth are known to be reduced, while permeability is increased following a period of fasting [23]. Mucosal atrophy could also be associated with disruption in the amount, or function of, digestive enzymes. In addition, mucosal oedema and reduced splanchnic blood flow may contribute to reduced absorption. Abnormal small intestinal motility may also be important [24] and accelerated transit would reduce the time for absorption. However, to date, small intestinal transit has not been formally examined in the critically ill population. It is possible that some critically ill patients have significant malabsorption and cannot be fed enterally. This needs further investigation and, if confirmed, methods to identify these patients clinically need to be developed.

In health and some disease states, GE is both determined by, and a determinant of, blood glucose concentrations [4]. This study found that slower GE was associated with a smaller increment in blood glucose in the healthy subjects, consistent with previous observations [25]. Although no relation between postprandial blood glucose concentrations and GE was demonstrated in the critically ill patients in this study, the postprandial increment in blood glucose was related to glucose absorption.

Hyperglycaemia occurs frequently in critical illness, has been attributed to insulin resistance, as well as abnormalities in the release and action of other regulatory hormones and the presence of inflammatory cytokines [8], and is associated with a worse clinical outcome [9,26]. The close relation between GE and glucose absorption suggests that, if GE is accelerated by the use of prokinetics, or if the stomach is bypassed and nutrient is placed directly into the small intestine, the rate of glucose absorption may be increased. This could have the undesirable effect of increasing blood glucose concentrations. However, it is unclear how important this effect is in patients receiving continuous infusions of enteral feeding because in this study the nutrient was delivered as a single naso-gastric bolus, which is likely to cause a greater increment in blood glucose concentration. This warrants further investigation.

Acute elevations in blood glucose concentration slow GE in healthy humans and patients with type 1 diabetes. Hyperglycaemia (about 15 mMol/l) markedly slows GE [5,27,28], but even changes in blood glucose concentrations within the normal postprandial range (4 to 8 mmol/l) can have a significant impact [29-31]. Consistent with these findings, this study found an inverse relation between baseline blood glucose concentrations and subsequent GE in the patients, such that higher blood glucose was associated with slower GE. The absence of a relation in healthy subjects is not surprising given that blood glucose concentrations were much lower (maximum 6.4 mMol/l). Hence, it is possible that in ICU patients glycaemia influences GE. However, the causes of delayed GE are likely to be multifactorial and the relative importance of changes in blood glucose concentrations is as yet unclear. Hyperglycaemia may also reduce the effect of prokinetic drugs such as erythromycin [32-35] and metoclopramide.

There are some limitations in this study which need to be considered when interpreting the results. The kinetics of 3-OMG absorption have never been validated in the critically ill population. It is possible that kinetic variables, such as the volume of distribution and renal clearance, may affect 3-OMG concentrations following ingestion. These effects are likely to vary between individuals and in the same individual over time. An increase in volume of distribution would reduce 3-OMG concentrations but it is unlikely that this could account for the marked reduction in 3-OMG concentrations observed in this study. Similarly, three patients in this study were receiving renal replacement therapy. It is not known how 3-OMG is cleared by dialysis and so the effect of this on the 3-OMG concentrations cannot be predicted.

Blood samples for the measurement of glucose and 3-OMG were taken from an arterial line in the patients and a venous line in healthy subjects. There is a difference in blood glucose concentrations between arterial and venous samples, but this difference is generally believed to be small [36,37]. As 3-OMG is not metabolised by tissues, there is unlikely to be a difference between arterial and venous samples, but this has not been documented.

The number of subjects recruited was relatively small. Nevertheless, highly significant differences were observed between healthy subjects and critically ill patients, suggesting that a study with greater numbers is unlikely to generate different results. However, there was a difference in the age and gender ratio between the two groups. In health, GE is probably slightly slower in pre-menopausal women than in age-matched men [38-40]. Interestingly, the largest study to date examining GE in critically ill patients suggests that gender has the opposite effect, in that women had a faster emptying rate [2], although this is not a consistent finding [41,42]. It is possible that normal hormonal effects are less evident in critically ill patients, because critical illness causes marked aberrations in hormonal activity so the gender effect on GE may be less important. It is also likely that other factors have a stronger influence on GE causing marked slowing in some cases and obscuring the more subtle hormonal effects. In this study, there was a greater proportion of women in the healthy group, which could have resulted in a slowing of GE in this cohort. However, the current study demonstrated slowed GE in critically ill patients compared with healthy controls. We may have shown a greater difference if we had included more males in the control group.

The effect of healthy ageing on GE is uncertain with inconsistent observations [43-49]. Extreme ageing is thought to be associated with a slowing of GE, which may reflect an increase in small intestinal nutrient feedback [50]. Studies on the elderly usually evaluate subjects in the age range 65 to 80 years. The age range of the critically ill patients recruited into this study was 28 to 79 years (median 63). Heyland and colleagues [2] reported a small, but significant slowing of GE with increasing age in a mixed critically ill cohort [2]. It is possible that age may have contributed to the delays in GE observed in this critically ill cohort; however, its importance is unclear and any effect is likely to be small. It should be noted that the effects of gender imbalance and age would have opposing effects on the GE in the two groups. It is also possible that the differences in age and gender balance may be the cause of reduced glucose absorption in the critically ill group, but this unlikely.

## Conclusions

This study suggests that the rate and extent of glucose absorption is markedly reduced in critical illness. GE influences the rate of glucose absorption, but does not account for the reduction in total absorption. The use of therapeutic agents to stimulate GE would, therefore, be expected to increase the rate of nutrient absorption in these patients. Factors other than slow GE also appear to limit absorption in critically ill patients and investigation into small intestinal abnormalities may identify reversible causes. Stimulation of GE with prokinetic agents may therefore not be expected to *normalise *glucose absorption and this warrants further investigation. The identification of patients with severely compromised absorption may allow more successful nutrient delivery by an alternative route.

## Key messages

• The rate and extent of glucose absorption is markedly reduced in critically ill patients.

• A close relation exists between glucose absorption and the rate of GE, such that slow GE was associated with impaired absorption during critical illness.

• In patients with normal GE, glucose absorption was still reduced.

• Abnormalities other than delayed GE contribute to impaired absorption in the critically ill.

## Abbreviations

3-OMG: 3-O-methyl glucose; AUC: area under the concentration curve; GE: gastric emptying; ICU: intensive care unit.

## Competing interests

The authors declare that they have no competing interests.

## Authors' contributions

MC, RF and MH were involved with study conception and design, data interpretation, statistical analysis and drafting of the manuscript. MB, KJ and BC were involved in study design and scintigraphic data acquisition and interpretation. AR provided technical support for studies and data acquisition. LB was involved in data acquisition, technical support, analysis and revision of the manuscript. GM and RB were involved in study design and performed the analysis of plasma 3-OMG using HPLC. All authors read and approved the final manuscript.
